# Converting from ECCE to SICS

**Published:** 2009-03

**Authors:** Nick Astbury

**Affiliations:** Consultant Ophthalmic Surgeon, Norfolk and Norwich University Hospital NHS Trust, Colney Lane, Norwich NR4 7UY, UK.

**8^th^ General Assembly of IAPB**

**Course 14:** Converting from ECCE to manual SICS

**Speakers:** RD Ravindran, Felipe Chiriboga, Reggie Seimon, Rainald Duerksen, Albrecht Hennig

**Figure:**
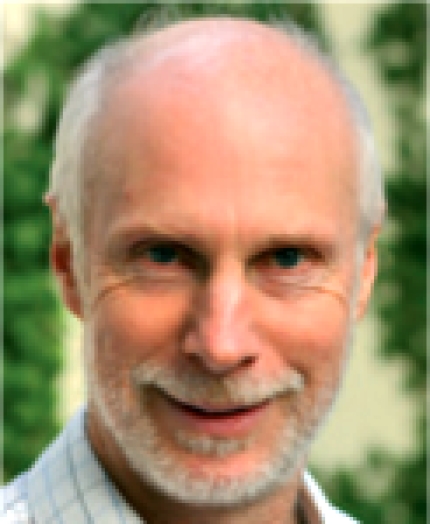


## Introduction

Cataract continues to be the cause of almost half the cases of blindness worldwide and the challenge to meet the needs and develop the required resources is as great as ever.

Cataract surgery has evolved from couching, first practised several thousand years ago, through intra- and extracapsular extraction (ECCE), to phacoemulsification. However, whatever the technique, the most important aspect is the outcome for patients. Today, the focus is more and more on excellence, which was one of the central themes of the 8^th^ General Assembly.

Good outcomes depend upon teamwork, appropriate surgical technique, and the expertise of the individual surgeon.

Outcomes using small incision ‘non-phaco’ techniques have been encouraging in settings where large volumes of surgery have been undertaken.^1^ Manual small incision cataract surgery (SICS) offers better outcomes than ECCE and provides results equally as good as phacoemulsification (‘phaco’), while being faster, cheaper, and less dependent on technology.^2^ It is therefore more appropriate for tackling cataract in low- and middle-income countries.

In developing countries, most surgeons are skilled in ECCE and would benefit from training to convert to SICS (see box). The availability of surgical training for SICS is becoming an increasingly important factor: indeed, ‘phaco’ surgeons have become more sophisticated in a technique that is inappropriate for tackling cataract on a global scale, and they are not best placed to teach small incision non-phaco techniques.

Increasingly, the focus of expertise in manual SICS is to be found in India, Pakistan, Nepal, Africa, or Latin America. The course on converting from ECCE to SICS featured four surgeons from Paraguay, Ecuador, Nepal, and India, all experts in manual SICS.

**Figure:**
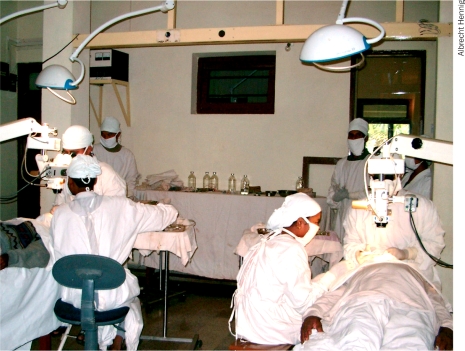
High-volume SICS cataract surgery. NEPAL

Albrecht Hennig

The speakers offered a glimpse of the variety of SICS techniques which have evolved in different parts of the world and discussed the best way to convert from ECCE to SICS.

## A variety of SICS techniques

### Paraguay

Rainald Duerksen described the manual small incision technique used at the Fundación Visión:

- 6–8 mm straight incision 3 mm from the limbus, with a 15 Beaver blade
- a three-plane, self-sealing, long tunnel (important to avoid iris prolapse)
- entry with a 2.75 or 3 mm crescent blade
- 5-6 mm capsulorhexis with either a 27 G needle or Utrata forceps
- hydrodissection and mobilisation of the nucleus
- injection of viscoelastic above and below the nucleus
- loop extraction of the nucleus
- cortical aspiration with a Simcoe cannula
- insertion of a polymethyl methacrylate intraocular lens (IOL).

Duerksen outlined the difficulties for trainees learning to convert to SICS. These included premature entry, an incomplete capsulorhexis and tearing of the posterior capsule, or creating a zonular dialysis whilst attempting to mobilise the nucleus.

### Ecuador

Felipe Chiriboga, from the Fundación Oftalmológica del Valle, also described a high-quality, low-cost, SICS technique suitable for all types of nuclei. Features of his technique included:

- topical and intracameral anaesthesia
- inverted smile incision to reduce astigmatism (which should be 1 mm wider when the surgeon is learning the technique)
- use of methylcellulose as a viscoelastic
- rotation of the nucleus with a Jaffe-Bechert nucleus rotator introduced through a 6 o'clock paracentesis
- fracture of the nucleus using an Akahoshi pre-chopper (Figure 1).
  **Figure 1:**
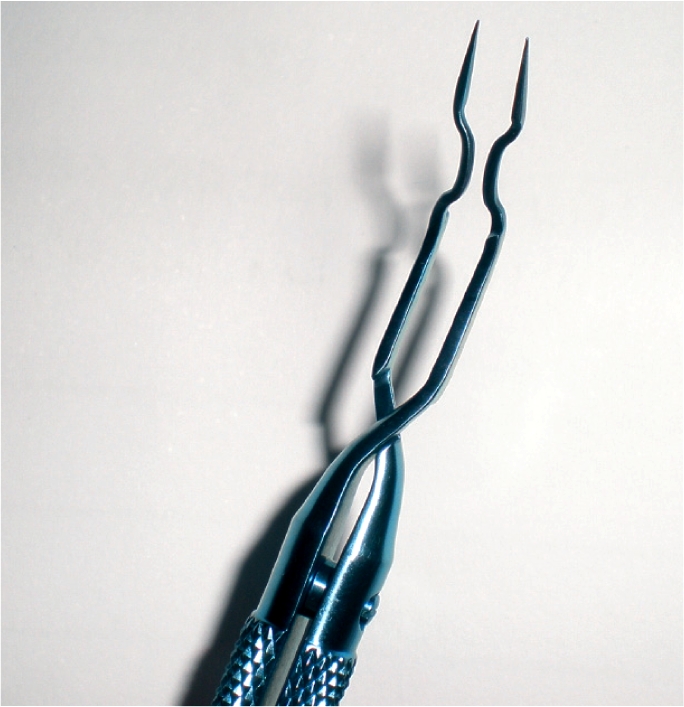
Akahoshi pre-chopper

Felipe Chiriboga

Chiriboga stressed the importance of using two different pre-choppers: a sharp one for harder nuclei and one with a wider point for softer cataracts.

### Nepal

**Figure 2:**
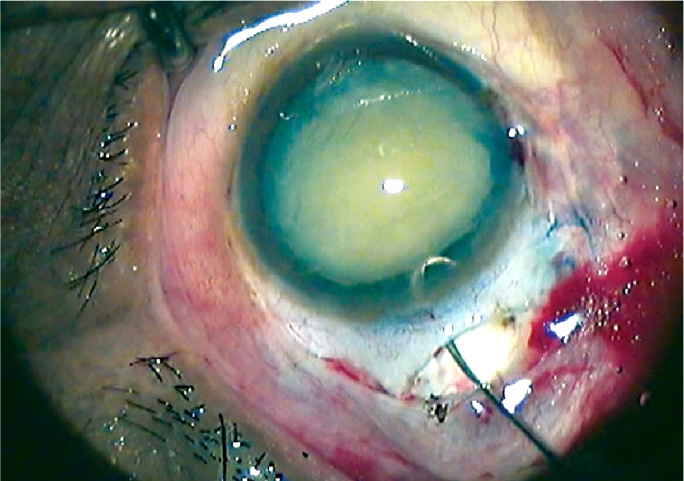
Nucleus extraction using a ‘fish-hook’

A ‘fish-hook’ made from a 30G needle (Figure 2) has now become well known as a cheap, reusable, and very effective instrument with which to remove the lens nucleus.^3^ Albrecht Hennig developed the technique in 1997 and over 375,000 operations have since been carried out in this manner at Lahan, in Nepal.

Albrect Hennig

Hennig stressed the advantages of the fish-hook technique:

- it is easy to learn
- the hook can be made from a bent 30G needle
- it requires a small tunnel.

### India

RD Ravindran from Aravind Eye Hospital, in Pondicherry, presented a SICS technique using an irrigating vectis, which will be described in more detail in the next section.

## Teaching the conversion from ECCE to SICS

### Barriers to training

Ravindran outlined some of the barriers to the conversion to SICS:

- lack of available training programmes
- reluctance of surgeons to change their technique
- increased difficulty for those who have not mastered ECCE beforehand.

He described particularly difficult stages in the learning process, which included:

- tunnel construction
- capsulotomy (or capsulorhexis)
- prolapsing the nucleus into the anterior chamber
- nucleus extraction
- removing stubborn cortex from under the incision.

### Teaching under supervision

Hennig stressed that learning to convert from ECCE should preferably be undertaken in a training centre, under supervision, and in a stepwise manner on selected patients (see Table 1 below).

**Table 1:** Stepwise conversion under supervision^a^

Stages of learning	Performed by trainer	Performed by trainee
**1**	Tunnel construction Nucleus removal	IOL insertion
**2**	Tunnel construction	Nucleus removal (through a smaller ab externo opening, using either a fish-hook or a vectis) IOL insertion
**3**	Supervision only	Tunnel construction (starting with smaller tunnels on immature cataracts) Nucleus removal IOL insertion

Experienced ECCE surgeons can also learn the transition to SICS by studying the techniques on videos and/or observing SICS surgery done by colleagues.

### Stepwise conversion to SICS: learning stages

Ravindran presented a scheme for converting to SICS by learning its steps in stages during the ECCE surgery.

**Stage 1** involves prolapsing the nucleus into the anterior chamber using a Sinskey hook, through a can-opener capsulotomy, and removing the nucleus with a vectis.

**Stage 2** involves vectis extraction through progressively smaller ECCE incisions, until an 8 mm sutured incision is achieved.

**Stage 3:** a sutured limbal tunnel is created with a crescent blade and keratome, the starting point being an 8 mm incision parallel to the limbus and 1 mm from clear cornea, which is then closed with three sutures after nucleus extraction using an irrigating vectis.

**Figure 3:**
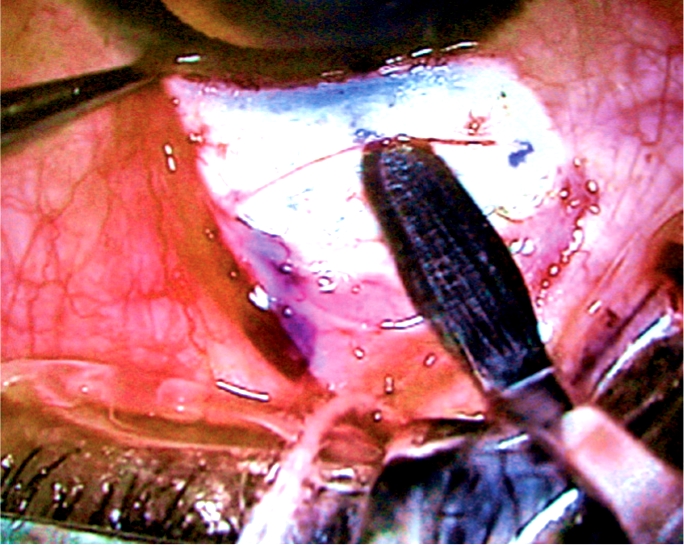
Frown incision

**Stage 4** is making a frown incision and tunnel (Figure 3). A fornix-based conjunctival flap is created, then a 1/3 to 1/2 thickness scleral groove (6–6.5 mm long), 1.5–2.0 mm from the limbus, and a tunnel extending 1–1.5 mm into clear cornea. A sideport is made in clear cornea, which is used for the capsulorhexis, to aspirate the cortex and to reform the anterior chamber.

RD Ravindran

**Stage 5** involves learning the capsulorhexis and nucleus extraction: a 6–6.5 mm capsulorhexis is done and the nucleus hydrodissected at 9 or 3 o'clock and a Sinskey hook used to wheel and mobilise it. An 8 × 5 mm irrigating vectis is placed below the nucleus and fluid injected when the nucleus has been pulled into the wound to create positive pressure. Then, with traction on the bridle suture, the nucleus is delivered applying pressure on the posterior lip of the wound. A large nucleus is fractured in the tunnel and the inner fragment reposited back and the hydroexpression repeated. After aspirating the cortex and checking the wound for retained material, the IOL is implanted.

Ravindran concluded that, with supervised stepwise conversion from ECCE and careful case selection, the transition to SICS is achievable with minimal complications. The box above offers useful tips for a successful conversion.

## Conclusion

As always, the video presentations made the surgery look straightforward, as is often the case when it is expertly performed, but the message that emerged from the course was that SICS can be undertaken using a variety of techniques but should be taught in a training centre in a stepwise fashion. The result is a procedure that is eminently suitable for tackling the cataract backlog without the use of hi-tech equipment and with excellent results.

**Advantages of SICS cersus ECCE^a^**

1. Shorter surgical time
2. Fewer instruments used
3. Cheaper
4. Faster visual recovery.

**Tips for a successful conversion to SICS**

1. Use stepwise learning under supervision
2. Start with simple cases
3. Avoid repetitive manoeuvres
4. Construct a tunnel of adequate size
5. Perform an adequate capsulotomy or capsulorhexis
6. Make liberal use of viscoelastic
7. Take time to master each step.
